# Association of Immune Nutrition Indices with the Risk of All-Cause Mortality and Cardiovascular Mortality in Patients with Heart Failure in the NHANES (1999–2018)

**DOI:** 10.31083/RCM25055

**Published:** 2025-01-09

**Authors:** Feifei Zhang, Yuetao Xie, Litian Liu, Huiliang Liu, Ohua Feng, Yingxiao Li, Yi Dang

**Affiliations:** ^1^Department of Cardiology Center, Hebei General Hospital, 050051 Shijiazhuang, Hebei, China; ^2^Department of Cardiology, Jingxing County Hospital, 050051 Shijiazhuang, Hebei, China

**Keywords:** heart failure, all-cause mortality, cardiovascular mortality, immune, nutrition, inflammatory

## Abstract

**Background:**

Heart failure (HF) remains a global challenge with disappointing long-term outcomes. Malnutrition is prevalent in patients with HF and disrupts the equilibrium of immune and inflammatory responses, resulting in further deterioration of the HF. Novel indicators emerge as immune nutrition indices, including the prognostic nutritional index (PNI), neutrophil-to-lymphocyte ratio (NLR), Controlling Nutritional Status (CONUT) score, and cholesterol-modified prognostic nutritional index (CPNI). This study examines the correlation between immune nutrition indices and all-cause and cardiovascular mortality in patients with HF.

**Methods:**

The data source for this study was the National Health and Nutrition Examination Survey (NHANES). A total of 1232 participants with HF were included. Weighted Cox proportional hazards models were employed to assess the independent association of different immune nutrition indices with mortality risk, alongside subgroup analyses and Kaplan–Meier survival curves. Restricted cubic spline analysis was utilized to clarify the detailed association between immune nutrition indices and hazard ratio (HR). A time-dependent receiver operating characteristic curve analysis was conducted to assess the predictive ability.

**Results:**

After full adjustments, PNI is independently related to all-cause mortality (HR = 0.94, 95% CI: 0.92–0.97) and cardiovascular mortality (HR = 0.94, 95% CI: 0.90–0.99). CPNI, CONUT, and NLR also showed an independent association with the prognosis of HF. Time-dependent receiver operating characteristic curve analysis indicated that PNI exhibited the highest predictive power for mortality among the CPNI, CONUT, and NLR indexes.

**Conclusions:**

Our study revealed that immune nutrition indicators, including CPNI, could predict all-cause mortality and cardiovascular mortality in the HF population. Compared with other indicators, PNI is the most effective predictor.

## 1. Introduction 

Heart failure (HF) is a terminal cardiovascular condition with a growing incidence worldwide, thereby presenting a challenge for global public health. As the population ages and the burden of chronic diseases increases, managing and treating HF becomes increasingly urgent [1]. Recent investigations have unveiled intricate links between HF and immune, nutritional, and inflammatory pathways. Malnutrition is prevalent in patients with HF, which could impair the ability to produce anti-inflammatory molecules and antioxidants, thereby weakening the immune system and making the body more susceptible to inflammatory insults, leading to further deterioration of the HF prognosis [2]. Assessing the nutritional and immunoinflammatory status in clinical practice shows potential for enhancing risk assessment, directing treatment strategies, and refining prognosis in managing HF.

Several immune nutrition indices have been increasingly utilized to provide valuable insights into disease severity, progression, and patient outcomes. Among these indices, the prognostic nutritional index (PNI), neutrophil-to-lymphocyte ratio (NLR), and Controlling Nutritional Status (CONUT) score have emerged as important prognostic tools [3, 4, 5]. The cholesterol-modified prognostic nutritional index (CPNI) has traditionally been assessed only in breast cancer patients to determine its impact on prognoses [6], with its value in predicting prognosis in HF patients yet to be confirmed. Furthermore, research on immune nutrition indices in HF has been limited by relatively small sample sizes and has mainly focused on hospitalized patients with more severe disease presentations. This makes it challenging to apply the findings to milder HF populations due to the disproportionate representation of more severe cases in previous studies.

This study explores the correlation between immune nutrition indices and the occurrence of all-cause and cardiovascular mortality in patients with HF. These data were derived from the NHANES (National Health and Nutrition Examination Survey) database. The goal is to recognize prognostic risk indicators (PNI, NLR, CONUT, and CPNI) for heart failure patients to facilitate early intervention and enhance patient outcomes. NHANES is a nationally representative cohort with diverse ethnicities and employs a complex, multistage probability sampling design. Therefore, findings from this database can be reliably extrapolated to real-world scenarios, enhancing the robustness of our conclusions. Compared to previous studies, we include a broader population and, for the first time, explore the significance of CPNI in predicting the prognosis of HF patients.

## 2. Methods

### 2.1 Data Source

The NHANES database, overseen by the National Center for Health Statistics (NCHS), is accessible to the public and maintains rigorous data collection and management standards. NHANES data were extracted using a detailed stratified, multistage, and complex sampling process. More information can be found in the NHANES Analytic Guidelines (https://wwwn.cdc.gov/nchs/nhanes/tutorials/default.aspx). This method involved gathering demographic details, conducting physical examinations, blood tests, and extensive surveys to provide an overview of the U.S. population. All participants in this survey provided informed consent in compliance with ethical standards.

### 2.2 Study Population

**Fig. 1.**
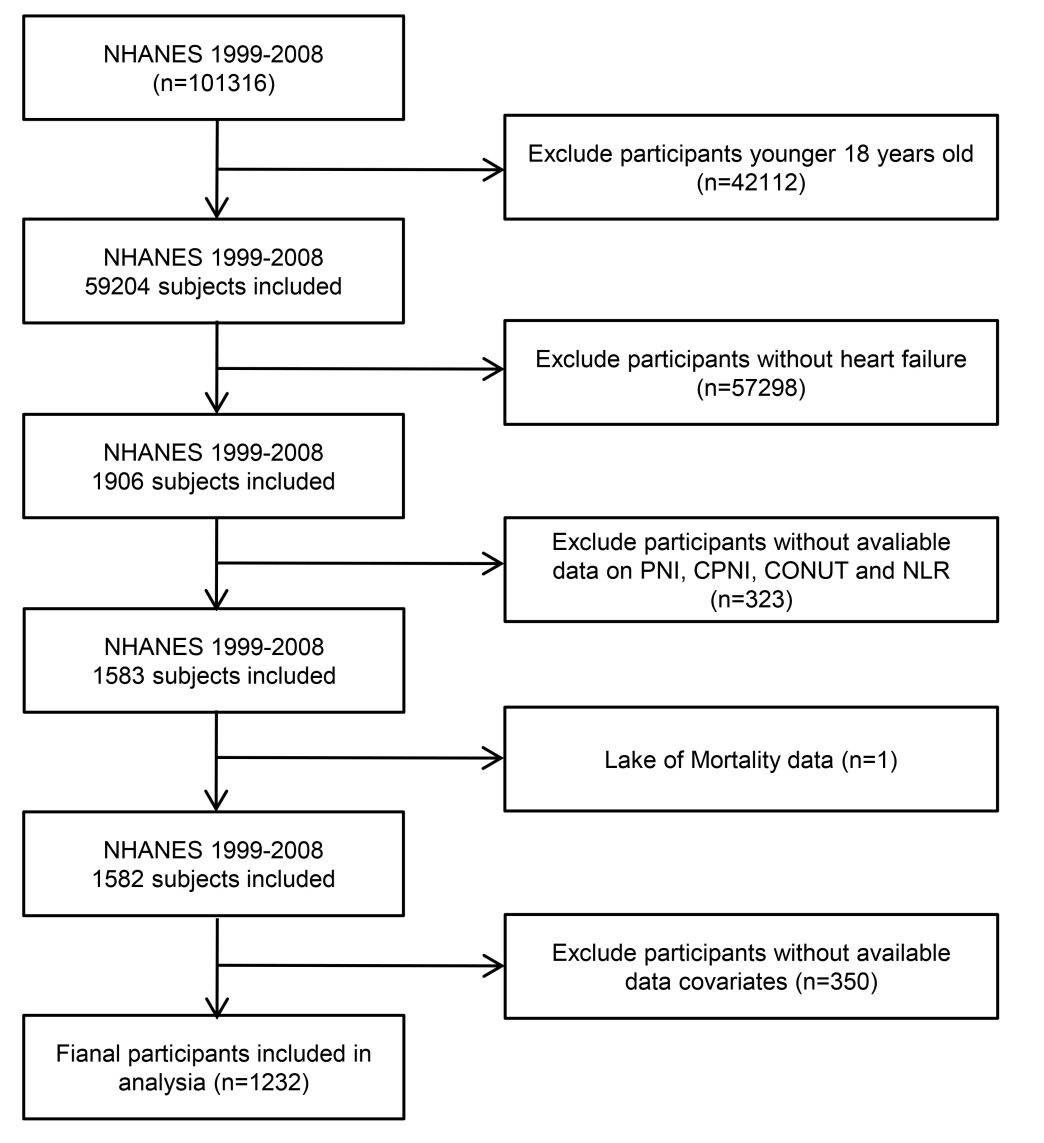
**Flowchart of the selection criteria for participants**. NHANES, National Health and Nutrition Examination Survey; PNI, prognostic nutritional index; CPNI, cholesterol-modified prognostic nutritional index; NLR, neutrophil-to-lymphocyte ratio; CONUT, controlling nutritional status.

A total of 101,316 enrolled participants were screened across ten consecutive NHANES cycles from 1999/2000 to 2017/2018. In the beginning, 42,112 participants under the age of 18 were excluded. Subsequently, 5729 participants without HF were removed. Further, 423 participants were excluded due to incomplete data for calculating the PNI, CPNI, CONUT, and NLR. Additionally, 1 participant was ineligible due to a lack of follow-up records. Finally, 350 participants were removed because their covariate information was incomplete. Following exclusion, 1232 patients with HF were included in our final analysis (Fig. 1). Consistent with previous studies [7, 8, 9], the confirmation of HF was determined using the questionnaire, which included the question, “have you ever been told that you have congestive heart failure?” Participants who responded “Yes” were recognized as individuals with diagnosed heart failure.

### 2.3 Assessment for Immune Nutrition Indices

The immune nutrition indices were calculated from serum albumin, total cholesterol level, and counts of lymphocytes and neutrophils. The PNI, CPNI, CONUT, and NLR calculation methods are presented in **Supplementary Table 1**. Blood samples are typically collected either on survey vehicles or at designated sampling sites, after which laboratory tests are processed. This procedure adheres to strict laboratory testing protocols. The Beckman Coulter counting and grading approach was used to obtain complete blood count (CBC) parameters. Serum albumin levels were determined using the dye bromcresol purple (BCP). An enzymatic method was used to measure cholesterol.

### 2.4 Covariates

The selection of covariates was guided by correlation logic and previously published literature. Participant characteristics included age, gender, race, education, family income to poverty ratio (PIR), marital status, body mass index (BMI), systolic blood pressure (SBP), diastolic blood pressure (DBP), smoke status, hemoglobin A1c (HbA1c), triglycerides (TG), total cholesterol (TC), uric acid, estimated glomerular filtration rate (eGFR), serum albumin, sodium, potassium, iron, hemoglobin, platelet count, neutrophils count, monocyte count, and presence of hypertension, coronary heart disease (CHD), stroke, cancer, asthma, and anemia. Race was classified into four categories. Education levels were divided into “less than high school”, “high school or equivalent”, and “college or higher”. The PIR was categorized as PIR <1.3 and PIR ≥1.3. Smoking was categorized into never (smoked less than 100 cigarettes in a lifetime), former (smoked more than 100 cigarettes in a lifetime but no smoking now), and current (actively smoking cigarettes daily or occasionally at the time of the survey). Diabetes was defined by any of the following criteria: a self-reported diagnosis by a doctor, use of insulin or oral glucose-lowering medications, plasma fasting glucose levels of 7.0 mmol/L or higher, or an HbA1c level of 6.5% or higher. A CONUT score of ≥2 points indicated malnutrition. The definitions of hypertension, CHD, stroke, cancer, asthma, and anemia were derived from self-reported questionnaire data. The eGFR was derived from the Chronic Kidney Disease Epidemiology Collaboration Equation (CKD-EPI Eq) based on serum creatinine [10]. Participants appointed to the mobile examination center (MEC) morning session were asked to fast for 9 hours. CBC tests were performed in the MEC, while other tests were completed in remote laboratories. The methods used include high-performance liquid chromatography (HPLC) for HbA1c and iron, colorimetric assay for TG and uric acid, and indirect (or diluted) ion selective electrode (I.S.E.) methodology for potassium and sodium.

### 2.5 Outcomes

Mortality data were extracted from the National Death Index. The Mortality Data Files link to NHANES. Currently, it includes mortality follow-up data updated up to 31 December 2019. The 10th edition of the International Classification of Diseases (ICD-10) was used to categorize the cause of death. Death from any cause was described as all-cause mortality. Cardiovascular mortality was specified as deaths caused by heart disease, including ICD-10 codes I00-I09, I11, I13, and I20-I51. The follow-up time for participants in the overall population was 73 (37, 122) months.

### 2.6 Statistical Analysis

Statistical evaluations adhered to the survey design characteristic of NHANES, utilizing suitable sampling weights. The Shapiro–Wilk test was used to evaluate the normality of continuous variables. Continuous variables were presented as the median and interquartile range and were compared using the Mann–Whitney U and Kruskal–Wallis tests between groups. Categorical variables are weighted percentages and were compared using the Chi-squared test. Weighted Cox regression models assess the relationships between immune nutrition indices and mortality across three different models, and restricted cubic splines (RCS) were performed further. Kaplan–Meier survival analysis and log-rank tests were conducted among groups. Additionally, the time-dependent receiver operating characteristic curve (ROC) was drawn to assess the accuracy of different immune nutrition indicators in forecasting survival outcomes at different times. Finally, stratified and interaction analyses were performed. Statistical significance was defined as *p* < 0.05. The R Project for Statistical Computing (version 4.3.3, R Foundation for Statistical Computing, Vienna, Austria) was used for analyses.

## 3. Results

### 3.1 Baseline Characteristics

A total of 1232 participants were included. The median age was 68.0 (58.0, 77.0) years; 55% were male. The median follow-up duration was 73 (37, 122) months. A total of 612 (47%) participants died, and 239 (17%) of these deaths were attributed to cardiovascular disease. The mortality group had a higher age, uric acid, potassium, neutrophils, monocytes, NLR, and CPNI and a lower BMI, DBP, eGFR, sodium, hemoglobin, platelet, and PNI than the survival group. Additionally, they tended to include a larger proportion of non-Hispanic Whites, were less married, possessed lower educational levels, and had a higher prevalence of asthma, anemia, and cancer (Table 1).

**Table 1.** **Baseline characteristics of participants**.

Characteristics		Overall	Alive	Died	*p*-value
		(n = 1232)	(n = 620)	(n = 612)	
Age (years)		68.0 (58.0, 77.0)	63.0 (51.0, 72.0)	74.0 (64.0, 80.0)	<0.001
Gender (%)					0.800
	Male	719 (55%)	352 (55%)	367 (54%)	
	Female	513 (45%)	268 (45%)	245 (46%)	
Race (%)					<0.001
	Mexican–American	118 (3.3%)	61 (4.1%)	57 (2.5%)	
	Non-Hispanic White	698 (75%)	291 (69%)	407 (82%)	
	Non-Hispanic Black	276 (12%)	166 (15%)	110 (9.4%)	
	Other	140 (9.0%)	102 (12%)	38 (6.2%)	
Married (%)		655 (57%)	356 (63%)	299 (51%)	<0.001
Education (%)					0.015
	College or higher	767 (69%)	411 (73%)	356 (64%)	
	High school or equivalent	242 (18%)	115 (17%)	127 (20%)	
	Less than high school	223 (13%)	94 (10%)	129 (16%)	
PIR (%)					>0.900
	PIR <1.3	485 (32%)	255 (32%)	230 (32%)	
	PIR ≥1.3	747 (68%)	365 (68%)	382 (68%)	
	BMI (kg/m^2^)	30 (26, 36)	31 (27, 37)	30 (26, 35)	0.001
	SBP (mmHg)	128 (115, 142)	127 (115, 140)	129 (114, 145)	0.200
	DBP (mmHg)	67 (59, 77)	70 (62, 80)	64 (56, 73)	<0.001
Smoke (%)					0.028
	Never	480 (38%)	247 (38%)	233 (38%)	
	Former	521 (42%)	241 (38%)	280 (45%)	
	Current	231 (21%)	132 (23%)	99 (17%)	
Asthma (%)		285 (26%)	168 (30%)	117 (21%)	0.006
Anemia (%)		137 (10%)	57 (7.7%)	80 (13%)	0.005
CHD (%)		529 (43%)	258 (41%)	271 (45%)	0.400
Stroke (%)		245 (20%)	105 (19%)	140 (21%)	0.400
Cancer (%)		267 (25%)	110 (22%)	157 (29%)	0.023
Hypertension (%)		955 (75%)	498 (77%)	457 (73%)	0.200
Diabetes (%)		562 (42%)	268 (39%)	294 (45%)	0.150
HbA1c (%)		5.80 (5.40, 6.50)	5.80 (5.40, 6.40)	5.90 (5.50, 6.60)	0.150
TG (mmol/L)		1.58 (1.11, 2.35)	1.59 (1.11, 2.35)	1.57 (1.11, 2.32)	0.700
TC (mmol/L)		4.55 (3.80, 5.43)	4.55 (3.80, 5.47)	4.60 (3.83, 5.38)	>0.900
Uric acid (mmol/L)		369 (297, 440)	357 (292, 422)	381 (303, 464)	<0.001
eGFR (mL/min/1.73 m^2^)		65 (45, 89)	75 (54, 101)	55 (36, 76)	<0.001
Iron (umol/L)		13.3 (10.0, 17.6)	13.6 (10.6, 17.7)	12.8 (9.7, 17.2)	0.067
Sodium (mmol/L)		139.00 (137.50, 141.00)	139.00 (138.00, 141.00)	139.00 (137.00, 141.00)	0.048
Potassium (mmol/L)		4.11 (3.90, 4.40)	4.10 (3.90, 4.30)	4.20 (3.90, 4.50)	<0.001
Neutrophils (10^9^/L)		4.50 (3.60, 5.60)	4.40 (3.50, 5.40)	4.64 (3.60, 5.80)	0.011
Monocyte (10^9^/L)		0.60 (0.50, 0.70)	0.60 (0.50, 0.70)	0.60 (0.50, 0.80)	0.032
Hemoglobin (g/dL)		13.90 (12.70, 14.90)	14.00 (13.10, 15.00)	13.60 (12.40, 14.80)	0.001
Platelet count (10^9^/L)		219 (180, 266)	221 (180, 264)	218 (179, 267)	0.600
PNI		50.5 (47.5, 54.5)	51.0 (48.0, 55.0)	50.0 (46.0, 53.5)	<0.001
CPNI		72 (66, 78)	71 (65, 77)	73 (67, 79)	<0.001
NLR		2.43 (1.75, 3.33)	2.22 (1.63, 3.00)	2.69 (1.94, 3.65)	<0.001
CONUT		1 (0, 2)	1 (0, 1)	1 (0, 1)	0.063
CONUT category (%)					0.067
	No malnutrition	767 (64%)	408 (67%)	359 (60%)	
	Malnutrition	465 (36%)	212 (33%)	253 (40%)	
Follow-up time (months)		73 (37, 122)	90 (47, 149)	57 (28, 93)	<0.001
Cardiovascular mortality (%)		239 (17%)	0 (0%)	239 (37%)	<0.001

Note: PIR, poverty income ratio; BMI, body mass index; SBP, systolic blood pressure; DBP, diastolic blood pressure; CHD, coronary heart disease; HbA1c, hemoglobin A1c; TG, triglycerides; TC, total cholesterol; eGFR, estimated glomerular filtration rate; PNI, prognostic nutritional index; CPNI, cholesterol-modified prognostic nutritional index; NLR, neutrophil-to-lymphocyte ratio; CONUT, controlling nutritional status. Data are presented as the median (25–75% interquartile range) or weighted percentage %.

Significant differences in age, educational level, DBP, smoke status, HbA1c, TG, TC, eGFR, iron, monocyte, hemoglobin, and the prevalence of anemia were observed after stratifying by the PNI quartile (*p* < 0.05). The PNI quartiles are 47, 50.5, and 54 (**Supplementary Table 2**). Additionally, significant differences in age, gender, marriage status, SBP, TC, eGFR, iron, monocyte, hemoglobin, and the prevalence of anemia were observed after stratifying by the CPNI quartile (*p* < 0.05). The CPNI quartiles are 66.2, 72.2, and 77.9 (**Supplementary Table 3**). Significant differences in age, gender, marriage status, SBP, TC, eGFR, iron, hemoglobin, and the prevalence of anemia were observed after stratifying by the quartile of the NLR (*p* < 0.05). The NLR quartiles are 1.67, 2.38, and 3.33 (**Supplementary Table 4**). Finally, significant differences in age, PIR, SBP, DBP, smoke status, TG, TC, eGFR, iron, potassium hemoglobin, platelet, and the prevalence of anemia, CHD, cancer, and diabetes were observed after stratifying by the median of the CONUT (*p* < 0.05). The median of CONUT is 1 (**Supplementary Table 5**).

### 3.2 Relationship between Immune Nutrition Indices and All-Cause Mortality in HF Populations

Weighted Cox regression analyzed the relationship between immune nutrition indices and all-cause and cardiovascular mortality. Model 1: unadjusted; Model 2: adjusted for age, gender, race, marital status, education, PIR, BMI, SBP, DBP, smoke, asthma, CHD, stroke, cancer, hypertension, and diabetes; Model 3: additional adjustment for HbA1c, TG, TC, uric acid, eGFR, iron, sodium, potassium, neutrophils, monocytes hemoglobin, and platelets based on Model 2. The variance inflation factor (VIF) shows no collinearity among variables (**Supplementary Table 6**).

**Fig. 2.**
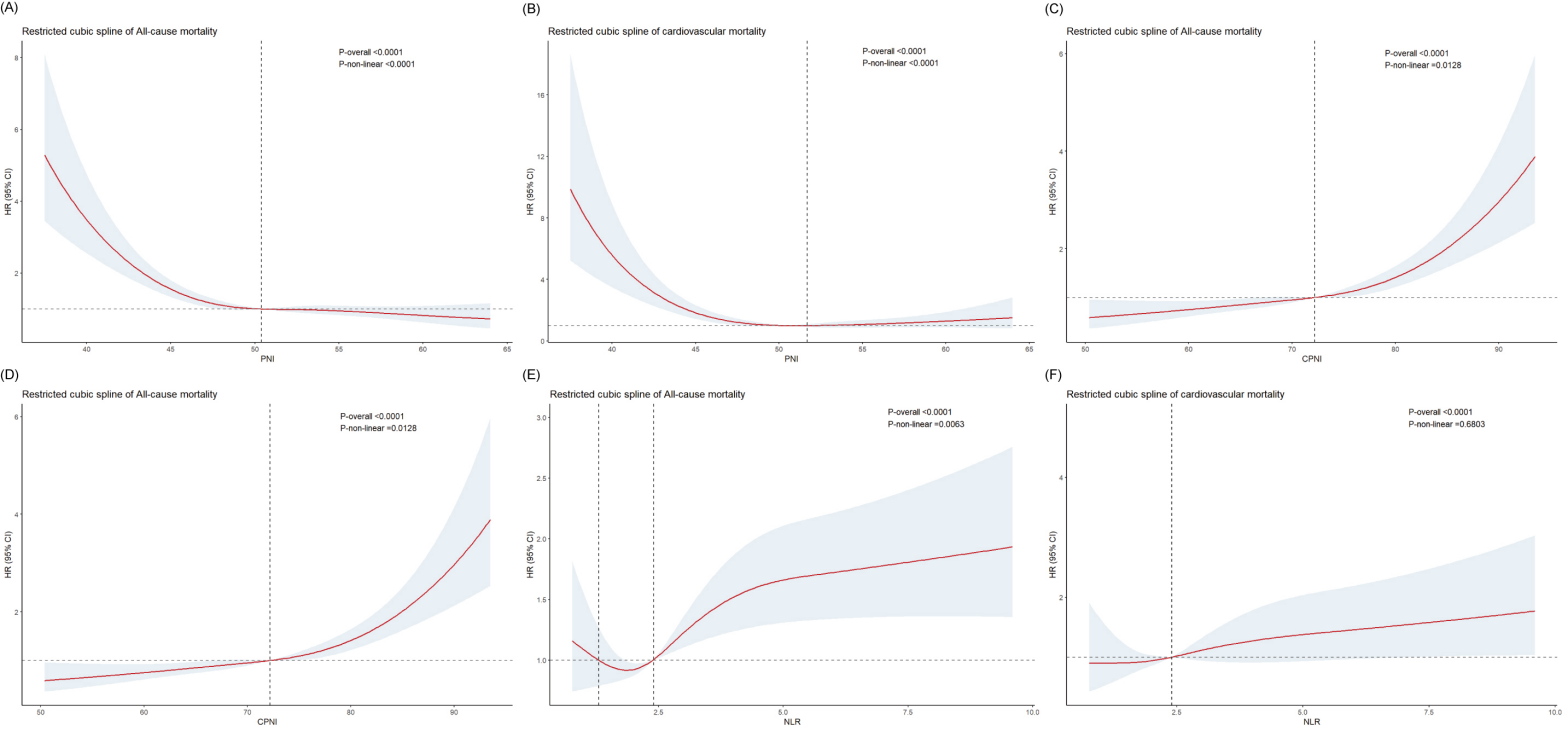
**Restricted cubic spline regression analysis**. (A,C,E) Non-linear relationship between PNI, CPNI, NLR, and all-cause mortality. (B,D) Non-linear relationship between PNI, CPNI, and cardiovascular mortality. (F) The linear relationship between NLR and cardiovascular mortality. Adjusted for age, gender, race, marriage, education, PIR group, BMI, SBP, DBP, smoke, asthma, anemia, CHD, stroke, cancer, hypertension, diabetes, HbA1c, triglycerides, total cholesterol, uric acid, eGFR, iron, sodium, potassium, neutrophils, monocytes, hemoglobin, and platelets. PNI, prognostic nutritional index; CPNI, cholesterol-modified prognostic nutritional index; NLR, neutrophil-to-lymphocyte ratio; PIR, poverty income ratio; BMI, body mass index; SBP, systolic blood pressure; DBP, diastolic blood pressure; CHD, coronary heart disease; HbA1c, hemoglobin A1c; eGFR, estimated glomerular filtration rate.

**Fig. 3.**
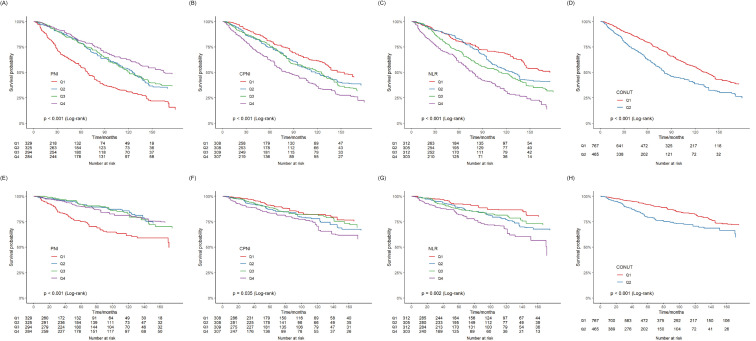
**Kaplan–Meier survival curve**. (A–D) Kaplan–Meier survival curve for all-cause mortality in populations with different PNI, CPNI, NLR, and CONUT levels. (E–H) Kaplan–Meier survival curve for cardiovascular mortality in populations with different PNI, CPNI, NLR, and CONUT levels. PNI, prognostic nutritional index; CPNI, cholesterol-modified prognostic nutritional index; NLR, neutrophil-to-lymphocyte ratio; CONUT, controlling nutritional status.

Model 1 (hazard ratio (HR) = 0.93, 95% CI: 0.90–0.95), Model 2 (HR = 0.94, 95% CI: 0.92–0.97), and Model 3 (HR = 0.94, 95% CI: 0.92–0.97). When evaluating PNI with different categories, the HRs for the Q2, Q3, and Q4 groups showed a significant decrease compared to the Q1 group across three established Cox models. Additionally, the trend *p*-values were below 0.001 for all models. Further analysis indicated that the CPNI, CONUT, and NLR were also independently related to all-cause mortality (Table 2). The RCS analysis showed a non-linear association between PNI, CPNI, NLR, and all-cause mortality (Fig. 2A,C,E). The relationship between PNI, CPNI, and cardiovascular mortality is non-linear (Fig. 2B,D), while the relationship between NLR and cardiovascular mortality is linear (Fig. 2F). Kaplan–Meier survival curves showed the different prevalences of all-cause mortality in the abovementioned groups, including PNI, CPNI, CONUT, and NLR. The survival rate was worse in the lower PNI group than in the higher PNI group. Participants with higher levels of CPNI, CONUT, and NLR showed a markedly reduced survival rate compared to those with lower levels (*p* for log-rank test <0.001) (Fig. 3).

**Table 2.** **HR (95% CIs) for all-cause mortality associated with immune nutrition indices**.

Characteristics		Model 1		Model 2		Model 3	
		HR (95% CI)	*p*-value	HR (95% CI)	*p*-value	HR (95% CI)	*p*-value
PNI		0.93 (0.90, 0.95)	<0.001	0.94 (0.92, 0.97)	<0.001	0.94 (0.92, 0.97)	<0.001
PNI quartiles							
	Q1 (≤47.00)	1.00 (Reference)	-	1.00 (Reference)	-	1.00 (Reference)	-
	Q2 (47.00–50.50)	0.49 (0.37, 0.66)	<0.001	0.56 (0.42, 0.75)	<0.001	0.61 (0.46, 0.81)	<0.001
	Q3 (50.50–54.00)	0.48 (0.37, 0.63)	<0.001	0.51 (0.39, 0.68)	<0.001	0.53 (0.39, 0.71)	<0.001
	Q4 (≥54.00)	0.36 (0.26, 0.48)	<0.001	0.50 (0.38, 0.66)	<0.001	0.50 (0.37, 0.68)	<0.001
*p* for trend			<0.001		<0.001		<0.001
CPNI		1.03 (1.02, 1.05)	<0.001	1.03 (1.02, 1.04)	<0.001	1.04 (1.02, 1.06)	<0.001
CPNI quartiles							
	Q1 (≤66.2)	1.00 (Reference)	-	1.00 (Reference)	-	1.00 (Reference)	-
	Q2 (62.2–72.2)	1.34 (0.99, 1.82)	0.062	1.24 (0.92, 1.69)	0.200	1.33 (0.97, 1.81)	0.072
	Q3 (72.2–77.9)	1.41 (1.04, 1.91)	0.029	1.24 (0.94, 1.64)	0.130	1.24 (0.90, 1.71)	0.200
	Q4 (≥77.9)	2.10 (1.53, 2.88)	<0.001	1.93 (1.40, 2.66)	<0.001	2.36 (1.63, 3.42)	<0.001
*p* for trend			<0.001		<0.001		<0.001
NLR		1.11 (1.05, 1.17)	<0.001	1.07 (1.02, 1.13)	0.005	1.06 (1.00, 1.12	0.034
NLR quartiles							
	Q1 (≤1.67)	1.00 (Reference)	-	1.00 (Reference)	-	1.00 (Reference)	-
	Q2 (1.67–2.38)	1.31 (0.92, 1.87)	0.140	1.00 (0.74, 1.33)	>0.900	0.93 (0.69, 1.26)	0.700
	Q3 (2.38–3.33)	1.73 (1.20, 2.50)	0.003	1.15 (0.82, 1.61)	0.400	0.99 (0.68, 1.43)	>0.900
	Q4 (≥3.33)	2.73 (1.87, 3.96)	<0.001	1.73 (1.22, 2.44)	0.002	1.43 (0.95, 2.15)	0.085
*p* for trend			<0.001		0.001		0.094
CONUT		1.28 (1.18, 1.40)	<0.001	1.15 (1.04, 1.26)	0.004	1.24 (1.10, 1.39)	<0.001
CONUT median							
	Q1 (≤1)	1.00 (Reference)	-	1.00 (Reference)	-	1.00 (Reference)	-
	Q2 (>1)	1.68 (1.36, 2.08)	<0.001	1.26 (1.01, 1.59)	0.042	1.28 (0.97, 1.70)	0.087
*p* for trend			<0.001		0.042		0.087

Model 1: unadjusted model. Model 2: adjusted for age, gender, race, marriage, education, PIR group, BMI, SBP, DBP, smoke, asthma, anemia, CHD, stroke, cancer, hypertension, and diabetes. Model 3: further adjustments from Model 2 were made for HbA1c, triglycerides, total cholesterol, uric acid, eGFR, iron, sodium, potassium, neutrophils, monocytes, hemoglobin, and platelets. PNI, prognostic nutritional index; CPNI, cholesterol-modified prognostic nutritional index; NLR, neutrophil-to-lymphocyte ratio; CONUT, controlling nutritional status; PIR, poverty income ratio; BMI, body mass index; SBP, systolic blood pressure; DBP, diastolic blood pressure; CHD, coronary heart disease; HbA1c, hemoglobin A1c; eGFR, estimated glomerular filtration rate; HR, hazard ratio.

### 3.3 Relationship between Immune Nutrition Indices and Cardiovascular Mortality in HF Populations

Model 1 (HR = 0.93, 95% CI: 0.89–0.97), Model 2 (HR = 0.94, 95% CI: 0.90–0.99), and Model 3 (HR = 0.94, 95% CI: 0.90–0.99). When evaluating PNI with different categories, compared to the Q1 group, the HRs showed a significant decrease among the Q2, Q3, and Q4 groups in three established Cox models. Furthermore, the trend *p*-values were less than 0.05. Further analysis also indicated that the CPNI, CONUT, and NLR were independently related to cardiovascular mortality (Table 3). The RCS analysis revealed a non-linear relationship between PNI, CPNI, and cardiovascular mortality and a linear correlation between NLR and cardiovascular mortality (Fig. 2). Kaplan–Meier survival curves showed the prevalence of cardiovascular mortality based on PNI, CPNI, CONUT, and NLR. The survival rate was worse in the lower PNI group than in the higher PNI group. Participants with higher levels of CPNI, CONUT, and NLR exhibited a significantly lower survival rate (*p* for log-rank test <0.001) (Fig. 3).

**Table 3.** **HR (95% CIs) for cardiovascular mortality associated with immune nutrition indices**.

Characteristics		Model 1		Model 2		Model 3	
		HR (95% CI)	*p*-value	HR (95% CI)	*p*-value	HR (95% CI)	*p*-value
PNI		0.93 (0.89, 0.97)	0.001	0.94 (0.90, 0.99)	0.012	0.94 (0.90, 0.99)	0.028
PNI quartiles							
	Q1 (≤47.00)	1.00 (Reference)	-	1.00 (Reference)	-	1.00 (Reference)	-
	Q2 (47.00–50.50)	0.34 (0.23, 0.51)	<0.001	0.35 (0.24, 0.52)	<0.001	0.37 (0.25, 0.55)	<0.001
	Q3 (50.50–54.00)	0.37 (0.25, 0.55)	<0.001	0.36 (0.24, 0.54)	<0.001	0.37 (0.24, 0.56)	<0.001
	Q4 (≥54.00)	0.39 (0.25, 0.62)	<0.001	0.54 (0.36, 0.83)	0.005	0.55 (0.34, 0.89)	0.015
*p* for trend			<0.001		0.008		0.020
CPNI		1.03 (1.02, 1.05)	0.008	1.02 (1.00, 1.05)	0.032	1.04 (1.00, 1.07)	0.028
CPNI quartiles							
	Q1 (≤66.2)	1.00 (Reference)	-	1.00 (Reference)	-	1.00 (Reference)	-
	Q2 (62.2–72.2)	1.42 (0.89, 2.25)	0.140	1.23 (0.75, 2.02)	0.400	1.30 (0.78, 2.18)	0.300
	Q3 (72.2–77.9)	1.24 (0.73, 2.08)	0.400	1.00 (0.60, 1.66)	>0.900	0.99 (0.55, 1.80)	>0.900
	Q4 (≥77.9)	1.95 (1.20, 3.19)	0.007	1.74 (1.01, 2.99)	0.044	2.26 (1.07, 4.77)	0.032
*p* for trend			0.020		0.090		0.073
NLR		1.11 (1.04, 1.17)	<0.001	1.07 (1.00, 1.14)	0.048	1.07 (0.98, 1.16)	0.020
NLR quartiles							
	Q1 (≤1.67)	1.00 (Reference)	-	1.00 (Reference)	-	1.00 (Reference)	-
	Q2 (1.67–2.38)	1.74 (0.99, 3.07)	0.056	1.31 (0.79, 2.18)	0.300	1.26 (0.73, 2.18)	0.400
	Q3 (2.38–3.33)	1.59 (0.87, 2.92)	0.130	1.01 (0.57, 1.76)	>0.900	0.87 (0.45, 1.68)	0.700
	Q4 (≥3.33)	2.94 (1.61, 5.35)	<0.001	1.82 (1.00, 3.29)	0.049	1.60 (0.82, 3.11)	0.200
*p* for trend			<0.001		0.010		0.330
CONUT		1.35 (1.19, 1.53)	<0.001	1.23 (1.07, 1.41)	0.004	1.35 (1.14, 1.60)	<0.001
CONUT median							
	Q1 (≤1)	1.00 (Reference)	-	1.00 (Reference)	-	1.00 (Reference)	-
	Q2 (>1)	1.90 (1.41, 2.54)	<0.001	1.47 (1.07, 2.01)	0.017	1.51 (1.05, 2.18)	0.027
*p* for trend			<0.001		0.017		0.030

Model 1: unadjusted model. Model 2: adjusted for age, gender, race, marriage, education, PIR group, BMI, SBP, DBP, smoke, asthma, anemia, CHD, stroke, cancer, hypertension, and diabetes. Model 3: further adjustments from Model 2 were made for HbA1c, triglycerides, total cholesterol, uric acid, eGFR, iron, sodium, potassium, neutrophils, monocytes, hemoglobin, and platelets. PNI, prognostic nutritional index; CPNI, cholesterol-modified prognostic nutritional index; NLR, neutrophil-to-lymphocyte ratio; CONUT, controlling nutritional status; HR, hazard ratio; CHD, coronary heart disease; BMI, body mass index; eGFR, estimated glomerular filtration rate; DBP, diastolic blood pressure; SBP, systolic blood pressure; HbA1c, hemoglobin A1c; PIR, poverty income ratio.

### 3.4 The Prognostic Ability Comparison of Immune Nutrition Indices

**Fig. 4.**
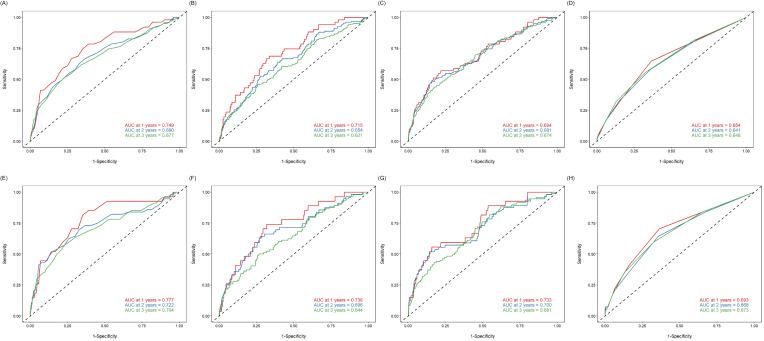
**Time-dependent ROC analysis**. (A,C,E,G) Time-dependent ROC analysis for all-cause mortality using PNI, CPNI, NLR, and CONUT in HF populations. (B,D,F,H) Time-dependent ROC analysis for cardiovascular mortality using PNI, CPNI, NLR, and CONUT in HF populations. HF, heart failure; PNI, prognostic nutritional index; CPNI, cholesterol-modified prognostic nutritional index; NLR, neutrophil-to-lymphocyte ratio; CONUT, controlling nutritional status; ROC, receiver operating characteristic curve; AUC, area under the curve.

**Fig. 5.**
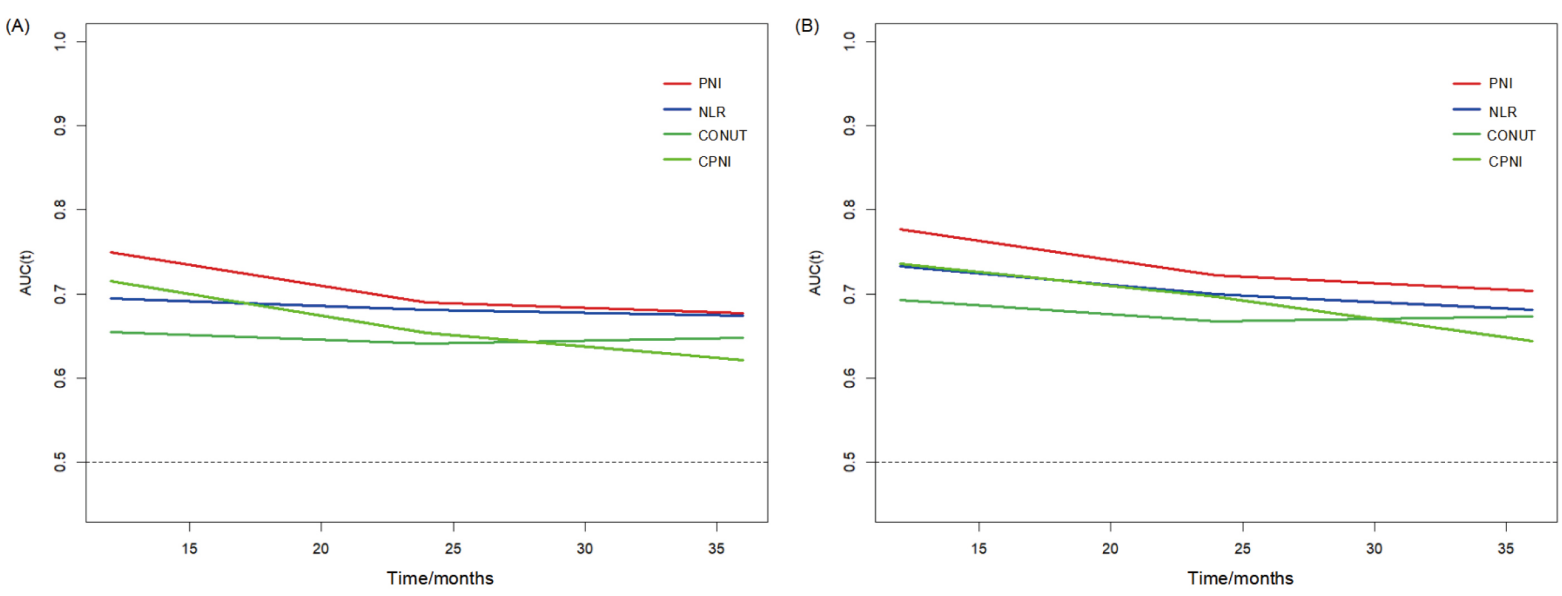
**The relationships of immune nutrition indicators mortality in HF populations**. (A) The prognostic ability comparison of PNI, CPNI, NLR, and CONUT for all-cause mortality in HF populations. (B) The prognostic ability comparison of PNI, CPNI, NLR, and CONUT for cardiovascular mortality in HF populations. HF, heart failure; PNI, prognostic nutritional index; CPNI, cholesterol-modified prognostic nutritional index; NLR, neutrophil-to-lymphocyte ratio; CONUT, controlling nutritional status; AUC, area under the curve.

A time-dependent ROC curve assesses the prognostic value of PNI, CPNI, CONUT, and NLR in HF participants. The results revealed that these indicators have predictive value for all-cause and cardiovascular deaths. However, their predictability has declined over time (Fig. 4). Compared with CPNI, CONUT, and NLR, PNI consistently had higher predictive power over a 3-year follow-up period (Fig. 5).

### 3.5 Subgroup and Interaction Analysis 

**Fig. 6.**
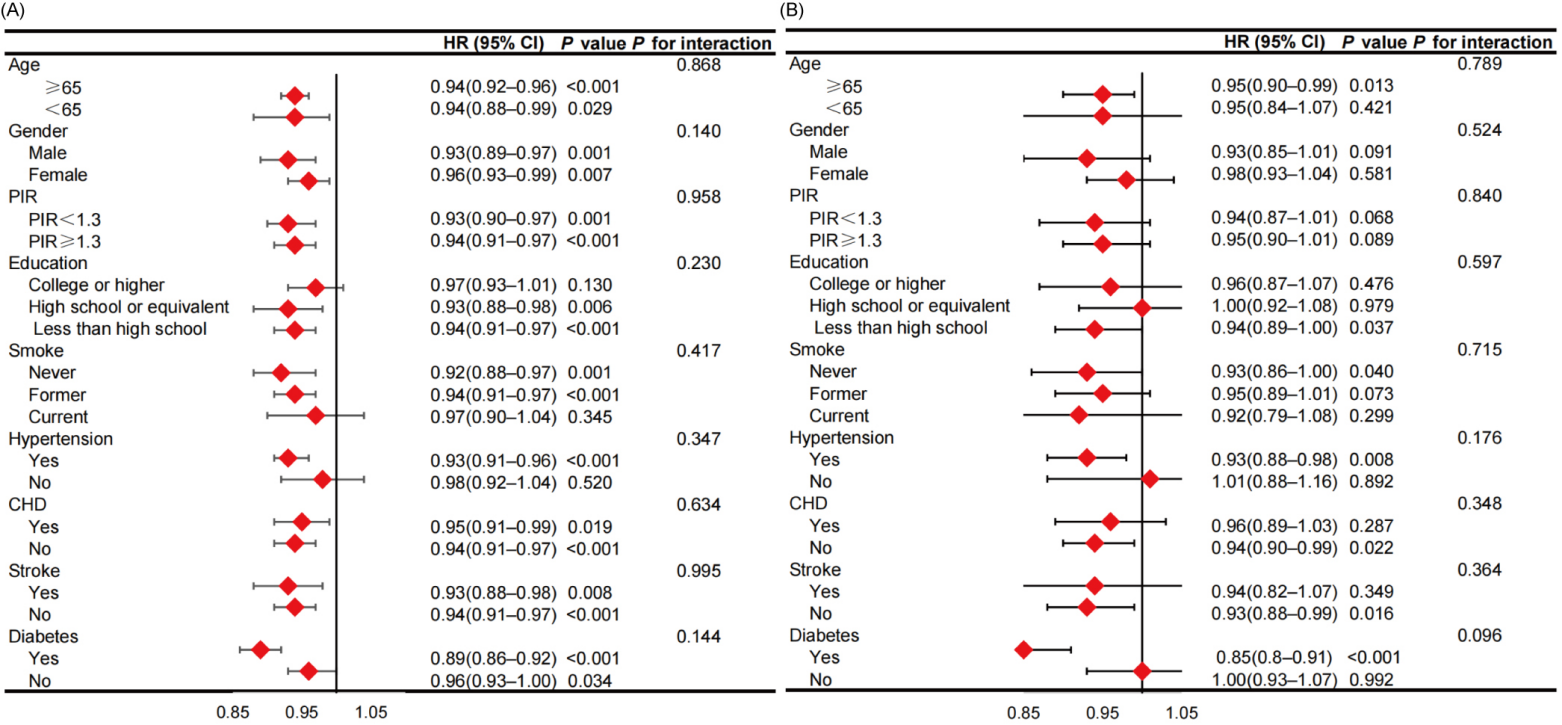
**Subgroup analysis and interaction analysis**. (A) Subgroup analysis for all-cause mortality. (B) Subgroup analysis for cardiovascular mortality. Adjusted for age, gender, race, marriage, education, PIR group, BMI, SBP, DBP, smoke, asthma, anemia, CHD, stroke, cancer, hypertension, diabetes, HbA1c, triglycerides, total cholesterol, uric acid, eGFR, iron, sodium, potassium, neutrophils, monocytes, hemoglobin, and platelets. PIR, poverty income ratio; CHD, coronary heart disease; HR, hazard ratio; BMI, body mass index; SBP, systolic blood pressure; DBP, diastolic blood pressure; HbA1c, hemoglobin A1c; eGFR, estimated glomerular filtration rate.

Subgroup analyses were conducted based on age, gender, education, PIR, hypertension, CHD, stroke, and diabetes. The findings indicate that PNI continues to be an independent protective factor for all-cause mortality, except in individuals with low education levels, current smokers, and those without hypertension. Conversely, its protective effect on cardiovascular mortality is evident only in individuals over 65 years old, with higher education levels, who have never smoked, and who have comorbid hypertension and diabetes but not stroke or CHD. Interaction analysis showed no interaction between PNI and these factors for all-cause or cardiovascular mortality (Fig. 6).

## 4. Discussion

This study examined the relationship between immune nutrition indices (PNI, CPNI, CONUT, and NLR) and the prognosis of HF populations to evaluate their prognostic value. All four indicators can be used as independent predictors of all-cause and cardiovascular death in HF. To our knowledge, we confirm the value of CPNI in evaluating the prognosis of HF for the first time. The time-dependent ROC revealed that PNI has the most effective predictability.

HF is still a global challenge, and clinical research remains focused on identifying prognostic factors for mortality. HF is not merely a cardiac issue but a complex systemic disease that could worsen the nutritional status of patients [11]. Furthermore, the resulting malnutrition may disturb the balance status of the immune and inflammation and aggravate the progression of HF, leading to a vicious circle [12, 13]. Our study indicates that the prevalence of malnutrition in the HF population, according to CONUT, was 36%. Notably, malnutrition was much more prevalent in the dead group.

The PNI comprehensively assesses patients’ nutritional and inflammatory status based on peripheral lymphocyte counts and serum albumin levels. Some previous studies have shown an association with prognosis in various malignancies, chronic inflammatory diseases, liver cirrhosis, type 2 diabetes, and chronic kidney disease [14, 15, 16, 17, 18]. Previous studies also explored whether lower PNI may reflect high mortality in acute decompensated HF patients with various left ventricular ejection fractions (LVEFs) [19, 20, 21, 22]. Additionally, it correlated with higher readmission rates and mortality in chronic HF [22, 23, 24]. Further, meta-analysis indicated that PNI could be an important indicator for risk stratification in the HF population [25]. Consistent with former results, our study showed that PNI was predictive of all-cause and cardiovascular death for HF. PNI has the best forecasting ability among CPNI, CONUT, and NLR.

CPNI is a modification of the PNI. We demonstrate for the first time the value of this composite indicator in the prognosis of HF. CPNI encompassed total cholesterol, albumin, and lymphocytes and was initially proposed as an independent predictor of breast cancer outcomes [6]. Albumin maintains nutrient reserves, supports immune functions, and regulates body fluids. Lymphocytes regulate the immune system by secreting cytokines and engaging in cytolytic activity. The cholesterol level in the blood is associated with oxidative stress and inflammation [26]. They are all involved in the pathophysiological processes of HF and affect disease progression. However, our findings do not support the cholesterol paradox in HF patients, which is the association of higher cholesterol levels with better survival [27]. We suggest that higher total cholesterol levels and lower albumin and lymphocyte levels are at higher risk of death in HF patients. Furthermore, Filipe verified that the cholesterol paradox may be attenuated in diabetic HF patients [28].

The CONUT score also incorporates the abovementioned three parameters: albumin, lymphocytes, and total cholesterol. Further, it is commonly used to assess nutritional status, with a higher CONUT score indicating malnutrition [17, 29, 30, 31]. Former studies have shown that a higher CONUT score is associated with unfavorable prognosis in CHD patients, leading to an increase in mortality and infection in HF patients [29, 32]. We also demonstrated the value of CONUT in predicting the prognosis of HF, although its predictive power was lower than that of CPNI.

NLR is used as a new additional inflammatory marker. Neutrophils, integral to the innate immune system, traditionally indicate the immune system’s inflammatory status, whereas lymphocytes regulate the immune system [21]. Fundamental research confirms that inflammation is indispensable in the pathogenesis of HF [33]. Previous clinical studies have certified that NLR could be a practical prognostic tool for risk stratification in HF populations [34]. However, these studies are limited by a small sample size. Our study extracted the HF population from the NHANES database. These individuals accurately reflected various social strata in the United States between 1998 and 2018. The total sample size amounted to 3,925,253. The ability to predict the prognosis of HF using NLR is inferior to PNI.

This study offers advantages due to its use of a nationally representative cohort of U.S. adult HF patients, improving the generalizability of the findings. Further, we first confirmed the value of CPNI for evaluating the prognosis of HF and compared it to different immune nutrition indices. However, several limitations should also be considered. This study was cross-sectional, thereby limiting its ability to elucidate the exact pathophysiological mechanisms underlying our findings. Additionally, this study did not take into consideration medication usage due to the limitations of the NHANES database, including the exclusion of angiotensin receptor-neprilysin inhibitor (ARNI) and sodium-dependent glucose transporters 2 (SGLT2) inhibitors, which have been proven to decrease mortality across the entire spectrum of heart failure. Furthermore, despite efforts to adjust for confounding factors, some unaccounted variables may still exist, such as ultrasound data consideration.

## 5. Conclusions

Our study revealed that immune nutrition indicators, including CPNI, could predict all-cause mortality and cardiovascular mortality in patients with HF. Compared with other indicators, PNI is the most effective predictor.

## Data Availability

The data supporting this study’s findings are available from the corresponding author upon reasonable request.

## Abbreviations

HF, heart failure; PIR, poverty income ratio; BMI, body mass index; SBP, systolic blood pressure; DBP, diastolic blood pressure; CHD, coronary heart disease; HbA1c, hemoglobin A1c; TG, triglycerides; TC, total cholesterol; eGFR, estimated glomerular filtration rate; PNI, prognostic nutritional index; CPNI, cholesterol-modified prognostic nutritional index; NLR, neutrophil-to-lymphocyte ratio; CONUT, controlling nutritional status.

## Supplementary Material

Supplementary material associated with this article can be found, in the online version, at https://doi.org/10.31083/RCM25055.
