# Supplementary material for: Association of Immune Nutrition Indices with the Risk of All-Cause Mortality and Cardiovascular Mortality in Patients with Heart Failure in the NHANES (1999–2018)
Source: Rev Cardiovasc Med. 2025 Jan 9;26(1):25055. doi: 10.31083/RCM25055 (PMC11759971; doi:10.31083/RCM25055)
Supplement: Supplementary file 1 [file 2153-8174-26-1-25055-s1.docx]

# Supplementary Tables

Supplementary Table 1. Calculation methods for each immune nutrition index.

| Parameters |  |  |  |  |
| --- | --- | --- | --- | --- |
| PNI | PNI = 5× lymphocyte counts (10^9^/L) + albumin (g/L) | | | |
| CPNI | CPNI = 4.8 × cholesterol (mmol/L) − 1.5×albumin (g/L) − 7.7×lymphocyte + 126 | | | |
| NLR | NLR = neutrophil counts (10^9^/L) /lymphocyte counts (10^9^/L) | | | |
| CONUT | CONUT = scores of albumins + scores of lymphocytes + scores of total cholesterol levels. A total score of ≥ 2 points indicates malnutrition. Albumin (g/L) > 35, 30–34, 25–29, and < 25; lymphocyte counts (10^9^/L) ≥ 1.6, 1.2–1.59, 0.8–1.19, and < 0.8; and total cholesterol levels (mmol/L) ≥ 180, 140–180, 100–139, and < 100 are assigned scored as 0, 2, 4, and 6 points, respectively. | | | |

Notes: PNI, the prognostic nutritional index; CPNI, the cholesterol-modified prognostic nutritional index; NLR, neutrophil-to-lymphocyte ratio; CONUT, the controlling nutritional status

Supplementary Table 2. Baseline characteristics of participants stratified by quartiles of PNI.

| **Characteristics** | **PNI** | | | |  |
| --- | --- | --- | --- | --- | --- |
|  | **Q1 (≤47.00)**  **(n=329)** | **Q2 (47.00-50.50)**  **(n=325)** | **Q3 (50.50-54.00)**  **(n=294)** | **Q4 (≥54.00)**  **(n=284)** | ***P* value** |
| **Age (years)** | 71.0 (63.0, 80.0) | 68.0 (60.0, 77.0) | 68.0 (57.2, 78.0) | 63.0 (52.0, 74.0) | <0.001 |
| **Gender (%)** |  |  |  |  | 0.056 |
| **Male** | 179 (51%) | 194 (53%) | 161 (52%) | 185 (63%) |  |
| **Female** | 150 (49%) | 131 (47%) | 133 (48%) | 99 (37%) |  |
| **Race (%)** |  |  |  |  | 0.042 |
| **Mexican American** | 22 (2.6%) | 33 (3.8%) | 31 (3.6%) | 32 (3.4%) |  |
| **Non-Hispanic White** | 180 (74%) | 192 (77%) | 167 (76%) | 159 (73%) |  |
| **Non-Hispanic Black** | 94 (17%) | 67 (11%) | 67 (13%) | 48 (9.5%) |  |
| **Other** | 33 (7.0%) | 33 (8.0%) | 29 (7.3%) | 45 (14%) |  |
| **Married (%)** | 158 (53%) | 176 (61%) | 161 (59%) | 160 (56%) | 0.3 |
| **Education (%)** |  |  |  |  | 0.027 |
| **College or higher** | 221 (73%) | 202 (71%) | 184 (70%) | 160 (62%) |  |
| **High school or equivalent** | 59 (15%) | 74 (20%) | 47 (14%) | 62 (22%) |  |
| **Less than high school** | 49 (12%) | 49 (8.5%) | 63 (16%) | 62 (15%) |  |
| **PIR (%)** |  |  |  |  | 0.3 |
| **PIR<1.3** | 119 (29%) | 116 (30%) | 129 (35%) | 121 (35%) |  |
| **PIR ≥ 1.3** | 210 (71%) | 209 (70%) | 165 (65%) | 163 (65%) |  |
| **BMI (kg/m^2^)** | 30 (26, 37) | 31 (26, 38) | 30 (26, 36) | 30 (27, 34) | 0.2 |
| **SBP (mmHg)** | 129 (113, 144) | 129 (115, 142) | 129 (117, 142) | 125 (115, 141) | 0.6 |
| **DBP (mmHg)** | 64 (56, 73) | 67 (59, 77) | 67 (58, 77) | 69 (62, 77) | <0.001 |
| **Smoke (%)** |  |  |  |  | 0.019 |
| **Never** | 118 (36%) | 146 (44%) | 119 (39%) | 97 (32%) |  |
| **Former** | 162 (49%) | 131 (40%) | 111 (37%) | 117 (41%) |  |
| **Current** | 49 (15%) | 48 (16%) | 64 (25%) | 70 (27%) |  |
| **Asthma (%)** | 85 (31%) | 75 (25%) | 59 (21%) | 66 (26%) | 0.3 |
| **Anemia (%)** | 62 (19%) | 32 (9.6%) | 24 (6.3%) | 19 (6.5%) | <0.001 |
| **CHD (%)** | 128 (40%) | 142 (45%) | 131 (39%) | 128 (46%) | 0.4 |
| **Stroke (%)** | 69 (21%) | 59 (21%) | 64 (20%) | 53 (19%) | >0.9 |
| **Cancer (%)** | 85 (30%) | 74 (28%) | 54 (23%) | 54 (20%) | 0.1 |
| **Hypertension (%)** | 262 (75%) | 263 (81%) | 215 (69%) | 215 (72%) | 0.075 |
| **Diabetes (%)** | 166 (45%) | 161 (45%) | 127 (42%) | 108 (34%) | 0.14 |
| **HbAc1 (%)** | 5.90 (5.40, 6.50) | 5.90 (5.50, 6.70) | 5.90 (5.50, 6.60) | 5.70 (5.30, 6.30) | 0.028 |
| **TG (mmol/L)** | 1.35 (1.01, 1.91) | 1.58 (1.01, 2.09) | 1.72 (1.20, 2.56) | 1.78 (1.18, 2.88) | <0.001 |
| **TC (mmol/L)** | 4.05 (3.44, 5.09) | 4.71 (3.96, 5.38) | 4.60 (3.98, 5.43) | 4.81 (4.14, 5.84) | <0.001 |
| **Uric acid (mmol/L)** | 375 (292, 458) | 369 (309, 440) | 363 (303, 461) | 363 (297, 428) | 0.7 |
| **eGFR (ml/min/1.73m^2^)** | 54 (34, 75) | 64 (45, 90) | 65 (48, 87) | 75 (55, 99) | <0.001 |
| **Iron (umol/L)** | 11.1 (8.1, 14.8) | 13.3 (10.2, 17.3) | 12.9 (10.1, 17.6) | 15.5 (12.3, 19.5) | <0.001 |
| **Sodium (mmol/L)** | 139.00 (137.00, 141.00) | 139.00 (138.00, 141.00) | 139.00 (137.99, 141.00) | 139.00 (137.00, 141.00) | 0.6 |
| **Potassium (mmol/L)** | 4.20 (3.90, 4.50) | 4.10 (3.90, 4.30) | 4.10 (3.90, 4.32) | 4.10 (3.90, 4.40) | 0.2 |
| **Neutrophils (10^9^/L)** | 4.50 (3.43, 5.77) | 4.20 (3.55, 5.20) | 4.56 (3.70, 5.70) | 4.65 (3.60, 5.50) | 0.12 |
| **Monocyte (10^9^/L)** | 0.60 (0.50, 0.70) | 0.60 (0.50, 0.70) | 0.60 (0.50, 0.80) | 0.60 (0.50, 0.80) | <0.001 |
| **Hemoglobin (g/dL)** | 12.90 (11.81, 13.90) | 13.70 (12.70, 14.80) | 13.94 (13.20, 15.00) | 14.70 (13.70, 15.50) | <0.001 |
| **Platelet count (10^9^/L)** | 209 (172, 274) | 207 (178, 249) | 226 (194, 274) | 233 (194, 264) | 0.005 |
| **Follow-up time (months)** | 49 (24, 89) | 68 (38, 115) | 80 (41, 123) | 88 (48, 148) | <0.001 |
| **All-cause mortality (%)** | 208 (64%) | 153 (42%) | 134 (44%) | 117 (38%) | <0.001 |
| **Cardiovascular mortality (%)** | 89 (27%) | 57 (12%) | 41 (14%) | 52 (17%) | <0.001 |

Note: PIR, poverty income ratio; BMI, body mass index; SBP, systolic blood pressure; DBP, diastolic blood pressure; CHD, coronary heart disease; HbAc1, glycated hemoglobin; TG, triglycerides; TC, total cholesterol; eGFR, estimated glomerular filtration rate; PNI, the prognostic nutritional index. Data is presented as the median (25-75% interquartile range) or weighted percentage %.

Supplementary Table 3. Baseline characteristics of participants stratified by quartiles of CPNI.

| **Characteristic** | **CPNI** | | | | ***P* value** |
| --- | --- | --- | --- | --- | --- |
|  | **Q1 (≤66.2)**  **(n=308)** | **Q2 (62.2-72.2)**  **(n=308)** | **Q2 (72.2-77.9)**  **(n=309)** | **Q4 (≥77.9)**  **(n=307)** |  |
| **Age (years)** | 65.0 (53.3, 74.8) | 69.0 (58.0, 77.7) | 68.0 (60.0, 77.0) | 69.2 (59.0, 80.0) | 0.004 |
| **Gender (%)** |  |  |  |  | <0.001 |
| **Male** | 214 (70%) | 178 (50%) | 178 (54%) | 149 (44%) |  |
| **Female** | 94 (30%) | 130 (50%) | 131 (46%) | 158 (56%) |  |
| **Race (%)** |  |  |  |  | 0.4 |
| **Mexican American** | 36 (3.8%) | 32 (3.7%) | 25 (2.3%) | 25 (3.6%) |  |
| **Non-Hispanic White** | 171 (73%) | 174 (76%) | 187 (78%) | 166 (74%) |  |
| **Non-Hispanic Black** | 53 (10%) | 71 (11%) | 67 (12%) | 85 (15%) |  |
| **Other** | 48 (12%) | 31 (8.5%) | 30 (7.7%) | 31 (7.6%) |  |
| **Married (%)** | 183 (63%) | 170 (58%) | 166 (60%) | 136 (48%) | 0.023 |
| **Education (%)** |  |  |  |  | 0.13 |
| **College or higher** | 176 (62%) | 197 (72%) | 191 (71%) | 203 (70%) |  |
| **High school or equivalent** | 65 (21%) | 54 (15%) | 69 (20%) | 54 (17%) |  |
| **Less than high school** | 67 (17%) | 57 (13%) | 49 (9.2%) | 50 (12%) |  |
| **PIR (%)** |  |  |  |  | 0.7 |
| **PIR<1.3** | 133 (36%) | 118 (31%) | 121 (30%) | 113 (32%) |  |
| **PIR ≥ 1.3** | 175 (64%) | 190 (69%) | 188 (70%) | 194 (68%) |  |
| **BMI (kg/m^2^)** | 30 (27, 35) | 30 (26, 36) | 30 (26, 37) | 31 (26, 37) | 0.7 |
| **SBP (mmHg)** | 124 (114, 139) | 127 (115, 141) | 129 (113, 143) | 133 (117, 146) | 0.006 |
| **DBP (mmHg)** | 67 (59, 77) | 66 (60, 77) | 67 (57, 78) | 67 (59, 77) | >0.9 |
| **Smoke (%)** |  |  |  |  | 0.2 |
| **Never** | 111 (34%) | 123 (38%) | 123 (38%) | 123 (41%) |  |
| **Former** | 132 (41%) | 122 (42%) | 131 (38%) | 136 (45%) |  |
| **Current** | 65 (25%) | 63 (20%) | 55 (24%) | 48 (14%) |  |
| **Asthma (%)** | 62 (22%) | 61 (21%) | 80 (27%) | 82 (33%) | 0.06 |
| **Anemia (%)** | 21 (6.9%) | 33 (8.8%) | 31 (9.3%) | 52 (17%) | 0.005 |
| **CHD (%)** | 147 (49%) | 136 (41%) | 143 (45%) | 103 (36%) | 0.06 |
| **Stroke (%)** | 59 (18%) | 63 (21%) | 54 (21%) | 69 (20%) | >0.9 |
| **Cancer (%)** | 54 (20%) | 68 (27%) | 79 (27%) | 66 (27%) | 0.4 |
| **Hypertension (%)** | 239 (74%) | 234 (74%) | 237 (72%) | 245 (79%) | 0.5 |
| **Diabetes (%)** | 139 (42%) | 139 (40%) | 140 (43%) | 144 (42%) | >0.9 |
| **HbAc1 (%)** | 5.80 (5.40, 6.40) | 5.90 (5.40, 6.60) | 5.90 (5.50, 6.50) | 5.70 (5.50, 6.50) | 0.5 |
| **TG (mmol/L)** | 1.58 (1.11, 2.38) | 1.61 (1.09, 2.57) | 1.57 (1.10, 2.24) | 1.58 (1.11, 2.19) | 0.8 |
| **TC (mmol/L)** | 4.11 (3.49, 4.68) | 4.24 (3.78, 5.07) | 4.66 (3.83, 5.30) | 5.56 (4.84, 6.18) | <0.001 |
| **Uric acid (mmol/L)** | 369 (297, 440) | 363 (297, 440) | 381 (315, 452) | 357 (292, 440) | 0.4 |
| **eGFR (ml/min/1.73m^2^)** | 71 (51, 94) | 63 (47, 91) | 64 (40, 86) | 61 (40, 85) | 0.016 |
| **Iron (umol/L)** | 14.3 (11.9, 18.6) | 13.6 (10.0, 17.9) | 12.5 (9.3, 16.7) | 12.0 (9.3, 17.0) | <0.001 |
| **Sodium (mmol/L)** | 139.00 (138.00, 141.00) | 139.00 (137.58, 141.00) | 139.00 (138.00, 141.00) | 139.00 (137.00, 141.00) | 0.5 |
| **Potassium (mmol/L)** | 4.20 (3.99, 4.40) | 4.10 (3.80, 4.39) | 4.10 (3.90, 4.40) | 4.20 (3.90, 4.40) | 0.3 |
| **Neutrophils (10^9^/L)** | 4.70 (3.70, 5.70) | 4.60 (3.70, 5.63) | 4.50 (3.60, 5.56) | 4.25 (3.40, 5.50) | 0.2 |
| **Monocyte (10^9^/L)** | 0.60 (0.50, 0.80) | 0.60 (0.50, 0.70) | 0.60 (0.50, 0.70) | 0.60 (0.50, 0.70) | <0.001 |
| **Hemoglobin (g/dL)** | 14.40 (13.44, 15.10) | 13.90 (12.90, 14.84) | 13.60 (12.54, 14.60) | 13.39 (12.20, 14.80) | <0.001 |
| **Platelet count (10^9^/L)** | 221 (192, 258) | 220 (178, 272) | 207 (179, 262) | 224 (178, 270) | 0.6 |
| **Follow-up time (months)** | 85 (46, 136) | 76 (39, 116) | 77 (41, 121) | 52 (28, 97) | <0.001 |
| **All-cause mortality (%)** | 125 (38%) | 146 (46%) | 160 (48%) | 181 (57%) | 0.006 |
| **Cardiovascular mortality (%)** | 49 (15%) | 58 (18%) | 58 (16%) | 74 (20%) | 0.4 |

Note: PIR, poverty income ratio; BMI, body mass index; SBP, systolic blood pressure; DBP, diastolic blood pressure; CHD, coronary heart disease; HbAc1, glycated hemoglobin; TG, triglycerides; TC, total cholesterol; eGFR, estimated glomerular filtration rate; PNI, the prognostic nutritional index. Data is presented as the median (25-75% interquartile range) or weighted percentage %.

Supplementary Table 4. Baseline characteristics of participants stratified by quartiles of NLR.

| **Characteristic** | **NLR** | | | | ***P* value** |
| --- | --- | --- | --- | --- | --- |
|  | **Q1 (≤1.67)**  **(n=308)** | **Q2 (1.67-2.38)**  **(n=308)** | **Q2 (2.38-3.33)**  **(n=309)** | **Q4 (≥3.33)**  **(n=307)** |  |
| **Age (years)** | 65.0 (53.3, 74.8) | 69.0 (58.0, 77.7) | 68.0 (60.0, 77.0) | 69.2 (59.0, 80.0) | 0.004 |
| **Gender (%)** |  |  |  |  | <0.001 |
| **Male** | 214 (70%) | 178 (50%) | 178 (54%) | 149 (44%) |  |
| **Female** | 94 (30%) | 130 (50%) | 131 (46%) | 158 (56%) |  |
| **Race (%)** |  |  |  |  | 0.4 |
| **Mexican American** | 36 (3.8%) | 32 (3.7%) | 25 (2.3%) | 25 (3.6%) |  |
| **Non-Hispanic White** | 171 (73%) | 174 (76%) | 187 (78%) | 166 (74%) |  |
| **Non-Hispanic Black** | 53 (10%) | 71 (11%) | 67 (12%) | 85 (15%) |  |
| **Other** | 48 (12%) | 31 (8.5%) | 30 (7.7%) | 31 (7.6%) |  |
| **Married (%)** | 183 (63%) | 170 (58%) | 166 (60%) | 136 (48%) | 0.023 |
| **Education (%)** |  |  |  |  | 0.13 |
| **College or higher** | 176 (62%) | 197 (72%) | 191 (71%) | 203 (70%) |  |
| **High school or equivalent** | 65 (21%) | 54 (15%) | 69 (20%) | 54 (17%) |  |
| **Less than high school** | 67 (17%) | 57 (13%) | 49 (9.2%) | 50 (12%) |  |
| **PIR (%)** |  |  |  |  | 0.7 |
| **PIR<1.3** | 133 (36%) | 118 (31%) | 121 (30%) | 113 (32%) |  |
| **PIR ≥ 1.3** | 175 (64%) | 190 (69%) | 188 (70%) | 194 (68%) |  |
| **BMI (kg/m^2^)** | 30 (27, 35) | 30 (26, 36) | 30 (26, 37) | 31 (26, 37) | 0.7 |
| **SBP (mmHg)** | 124 (114, 139) | 127 (115, 141) | 129 (113, 143) | 133 (117, 146) | 0.006 |
| **DBP (mmHg)** | 67 (59, 77) | 66 (60, 77) | 67 (57, 78) | 67 (59, 77) | >0.9 |
| **Smoke (%)** |  |  |  |  | 0.2 |
| **Never** | 111 (34%) | 123 (38%) | 123 (38%) | 123 (41%) |  |
| **Former** | 132 (41%) | 122 (42%) | 131 (38%) | 136 (45%) |  |
| **Current** | 65 (25%) | 63 (20%) | 55 (24%) | 48 (14%) |  |
| **Asthma (%)** | 62 (22%) | 61 (21%) | 80 (27%) | 82 (33%) | 0.06 |
| **Anemia (%)** | 21 (6.9%) | 33 (8.8%) | 31 (9.3%) | 52 (17%) | 0.005 |
| **CHD (%)** | 147 (49%) | 136 (41%) | 143 (45%) | 103 (36%) | 0.06 |
| **Stroke (%)** | 59 (18%) | 63 (21%) | 54 (21%) | 69 (20%) | >0.9 |
| **Cancer (%)** | 54 (20%) | 68 (27%) | 79 (27%) | 66 (27%) | 0.4 |
| **Hypertension (%)** | 239 (74%) | 234 (74%) | 237 (72%) | 245 (79%) | 0.5 |
| **Diabetes (%)** | 139 (42%) | 139 (40%) | 140 (43%) | 144 (42%) | >0.9 |
| **HbAc1 (%)** | 5.80 (5.40, 6.40) | 5.90 (5.40, 6.60) | 5.90 (5.50, 6.50) | 5.70 (5.50, 6.50) | 0.5 |
| **TG (mmol/L)** | 1.58 (1.11, 2.38) | 1.61 (1.09, 2.57) | 1.57 (1.10, 2.24) | 1.58 (1.11, 2.19) | 0.8 |
| **TC (mmol/L)** | 4.11 (3.49, 4.68) | 4.24 (3.78, 5.07) | 4.66 (3.83, 5.30) | 5.56 (4.84, 6.18) | <0.001 |
| **Uric acid (mmol/L)** | 369 (297, 440) | 363 (297, 440) | 381 (315, 452) | 357 (292, 440) | 0.4 |
| **eGFR (ml/min/1.73m^2^)** | 71 (51, 94) | 63 (47, 91) | 64 (40, 86) | 61 (40, 85) | 0.016 |
| **Iron (umol/L)** | 14.3 (11.9, 18.6) | 13.6 (10.0, 17.9) | 12.5 (9.3, 16.7) | 12.0 (9.3, 17.0) | <0.001 |
| **Sodium (mmol/L)** | 139.00 (138.00, 141.00) | 139.00 (137.58, 141.00) | 139.00 (138.00, 141.00) | 139.00 (137.00, 141.00) | 0.5 |
| **Potassium (mmol/L)** | 4.20 (3.99, 4.40) | 4.10 (3.80, 4.39) | 4.10 (3.90, 4.40) | 4.20 (3.90, 4.40) | 0.3 |
| **Neutrophils (10^9^/L)** | 4.70 (3.70, 5.70) | 4.60 (3.70, 5.63) | 4.50 (3.60, 5.56) | 4.25 (3.40, 5.50) | 0.2 |
| **Monocyte (10^9^/L)** | 0.60 (0.50, 0.80) | 0.60 (0.50, 0.70) | 0.60 (0.50, 0.70) | 0.60 (0.50, 0.70) | <0.001 |
| **Hemoglobin (g/dL)** | 14.40 (13.44, 15.10) | 13.90 (12.90, 14.84) | 13.60 (12.54, 14.60) | 13.39 (12.20, 14.80) | <0.001 |
| **Platelet count (10^9^/L)** | 221 (192, 258) | 220 (178, 272) | 207 (179, 262) | 224 (178, 270) | 0.6 |
| **Follow-up time (months)** | 85 (46, 136) | 76 (39, 116) | 77 (41, 121) | 52 (28, 97) | <0.001 |
| **All-cause mortality (%)** | 125 (38%) | 146 (46%) | 160 (48%) | 181 (57%) | 0.006 |
| **Cardiovascular mortality (%)** | 49 (15%) | 58 (18%) | 58 (16%) | 74 (20%) | 0.400 |

Note: PIR, poverty income ratio; BMI, body mass index; SBP, systolic blood pressure; DBP, diastolic blood pressure; CHD, coronary heart disease; HbAc1, glycated hemoglobin; TG, triglycerides; TC, total cholesterol; eGFR, estimated glomerular filtration rate; PNI, the prognostic nutritional index. Data is presented as the median (25-75% interquartile range) or weighted percentage %.

Supplementary Table 5. Baseline characteristics of participants stratified by quartiles of COUNT

| **Characteristic** | **COUNT** | | ***P* value** |
| --- | --- | --- | --- |
|  | **Q1 (≤1)**  **(n=767)** | **Q1 (>1)**  **(n=465)** |  |
| **Age (years)** | 65.0 (55.0, 76.0) | 72.0 (64.0, 79.0) | <0.001 |
| **Gender (%)** |  |  | 0.062 |
| **Male** | 415 (52%) | 304 (59%) |  |
| **Female** | 352 (48%) | 161 (41%) |  |
| **Race (%)** |  |  | 0.6 |
| **Mexican American** | 83 (3.6%) | 35 (2.9%) |  |
| **Non-Hispanic White** | 427 (74%) | 271 (77%) |  |
| **Non-Hispanic Black** | 172 (12%) | 104 (12%) |  |
| **Other** | 85 (9.7%) | 55 (7.9%) |  |
| **Married (%)** | 402 (57%) | 253 (59%) | 0.5 |
| **Education (%)** |  |  | 0.058 |
| **College or higher** | 458 (67%) | 309 (73%) |  |
| **High school or equivalent** | 159 (20%) | 83 (15%) |  |
| **Less than high school** | 150 (13%) | 73 (12%) |  |
| **PIR (%)** |  |  | 0.007 |
| **PIR<1.3** | 327 (35%) | 158 (27%) |  |
| **PIR ≥ 1.3** | 440 (65%) | 307 (73%) |  |
| **BMI (kg/m^2^)** | 31 (27, 36) | 30 (25, 35) | 0.075 |
| **SBP (mmHg)** | 129 (116, 143) | 126 (111, 141) | 0.03 |
| **DBP (mmHg)** | 69 (61, 78) | 64 (56, 72) | <0.001 |
| **Smoke (%)** |  |  | 0.002 |
| **Never** | 302 (39%) | 178 (37%) |  |
| **Former** | 296 (38%) | 225 (49%) |  |
| **Current** | 169 (24%) | 62 (15%) |  |
| **Asthma (%)** | 191 (27%) | 94 (24%) | 0.5 |
| **Anemia (%)** | 63 (7.2%) | 74 (16%) | <0.001 |
| **CHD (%)** | 317 (39%) | 212 (49%) | 0.009 |
| **Stroke (%)** | 142 (18%) | 103 (23%) | 0.11 |
| **Cancer (%)** | 145 (23%) | 122 (31%) | 0.016 |
| **Hypertension (%)** | 592 (75%) | 363 (74%) | 0.7 |
| **Diabetes (%)** | 322 (39%) | 240 (47%) | 0.015 |
| **HbAc1 (%)** | 5.80 (5.50, 6.51) | 5.90 (5.40, 6.50) | 0.8 |
| **TG (mmol/L)** | 1.78 (1.22, 2.56) | 1.32 (0.91, 1.91) | <0.001 |
| **TC (mmol/L)** | 5.02 (4.37, 5.79) | 3.60 (3.26, 4.26) | <0.001 |
| **Uric acid (mmol/L)** | 369 (303, 434) | 375 (292, 458) | 0.2 |
| **eGFR (ml/min/1.73m^2^)** | 71 (50, 93) | 54 (36, 76) | <0.001 |
| **Iron (umol/L)** | 13.8 (10.5, 18.3) | 12.2 (9.3, 16.3) | <0.001 |
| **Sodium (mmol/L)** | 139.00 (138.00, 141.00) | 139.00 (137.00, 141.00) | 0.5 |
| **Potassium (mmol/L)** | 4.10 (3.90, 4.38) | 4.20 (3.90, 4.50) | 0.024 |
| **Neutrophils (10^9^/L)** | 4.50 (3.60, 5.60) | 4.60 (3.50, 5.60) | 0.8 |
| **Monocyte (10^9^/L)** | 0.60 (0.50, 0.70) | 0.60 (0.50, 0.70) | 0.14 |
| **Hemoglobin (g/dL)** | 14.10 (13.10, 15.10) | 13.30 (12.11, 14.50) | <0.001 |
| **Platelet count (10^9^/L)** | 232 (194, 272) | 200 (167, 256) | <0.001 |
| **Follow-up time (months)** | 83 (43, 134) | 55 (28, 94) | <0.001 |
| **All-cause mortality (%)** | 359 (44%) | 253 (52%) | 0.067 |
| **Cardiovascular mortality (%)** | 131 (15%) | 108 (21%) | 0.055 |

Note: PIR, poverty income ratio; BMI, body mass index; SBP, systolic blood pressure; DBP, diastolic blood pressure; CHD, coronary heart disease; HbAc1, glycated hemoglobin; TG, triglycerides; TC, total cholesterol; eGFR, estimated glomerular filtration rate; PNI, the prognostic nutritional index. Data is presented as the median (25-75% interquartile range) or weighted percentage %.

Supplementary Table 6. Collinearity Test Results for Each Variable

|  | **GVIF** | **Df** | **GVIF^(1/(2*Df))** |
| --- | --- | --- | --- |
| **PNI** | 2.36 | 1 | 1.54 |
| **Age** | 3.72 | 1 | 1.93 |
| **Gender** | 3.37 | 1 | 1.84 |
| **Race** | 7.5 | 3 | 1.4 |
| **Married** | 2.55 | 1 | 1.6 |
| **Education** | 3.31 | 2 | 1.35 |
| **PIR** | 2.16 | 1 | 1.47 |
| **BMI** | 2.36 | 1 | 1.54 |
| **SBP** | 2.31 | 1 | 1.52 |
| **DBP** | 2.12 | 1 | 1.46 |
| **Smoke** | 5.39 | 2 | 1.52 |
| **Asthma** | 2.03 | 1 | 1.43 |
| **Anemia** | 2.73 | 1 | 1.65 |
| **CHD** | 1.9 | 1 | 1.38 |
| **Stroke** | 1.97 | 1 | 1.4 |
| **Cancer** | 1.84 | 1 | 1.36 |
| **Hypertension** | 1.52 | 1 | 1.23 |
| **Diabetes** | 2.88 | 1 | 1.7 |
| **HBA1c** | 2.46 | 1 | 1.57 |
| **TG** | 2.25 | 1 | 1.5 |
| **TC** | 2.03 | 1 | 1.42 |
| **Neutrophils** | 2.89 | 1 | 1.7 |
| **Monocyte** | 2.69 | 1 | 1.64 |
| **Uric acid** | 2.27 | 1 | 1.51 |
| **eGFR** | 2.94 | 1 | 1.72 |
| **Iron** | 2 | 1 | 1.41 |
| **Sodium** | 2.29 | 1 | 1.51 |

Note: PIR, poverty income ratio; BMI, body mass index; SBP, systolic blood pressure; DBP, diastolic blood pressure; CHD, coronary heart disease; HbAc1, glycated hemoglobin; TG, triglycerides; TC, total cholesterol; eGFR, estimated glomerular filtration rate; PNI, the prognostic nutritional index.
